# Effects of the cannabinoid CB_1_ agonist ACEA on salicylate ototoxicity, hyperacusis and tinnitus in guinea pigs

**DOI:** 10.1016/j.heares.2017.10.012

**Published:** 2017-12

**Authors:** Joel I. Berger, Ben Coomber, Samantha Hill, Steve P.H. Alexander, William Owen, Alan R. Palmer, Mark N. Wallace

**Affiliations:** a Medical Research Council Institute of Hearing Research, School of Medicine, The University of Nottingham, University Park, Nottingham, NG7 2RD, United Kingdom; b School of Life Sciences, Medical School, The University of Nottingham, Nottingham, NG7 2UH, United Kingdom

**Keywords:** Tinnitus, Cannabinoids, Chronic recording, Auditory cortex, Treatment, Salicylate

## Abstract

Cannabinoids have been suggested as a therapeutic target for a variety of brain disorders. Despite the presence of their receptors throughout the auditory system, little is known about how cannabinoids affect auditory function. We sought to determine whether administration of arachidonyl-2′-chloroethylamide (ACEA), a highly-selective CB_1_ agonist, could attenuate a variety of auditory effects caused by prior administration of salicylate, and potentially treat tinnitus. We recorded cortical resting-state activity, auditory-evoked cortical activity and auditory brainstem responses (ABRs), from chronically-implanted awake guinea pigs, before and after salicylate + ACEA. Salicylate-induced reductions in click-evoked ABR amplitudes were smaller in the presence of ACEA, suggesting that the ototoxic effects of salicylate were less severe. ACEA also abolished salicylate-induced changes in cortical alpha band (6–10 Hz) oscillatory activity. However, salicylate-induced increases in cortical evoked activity (suggestive of the presence of hyperacusis) were still present with salicylate + ACEA. ACEA administered alone did not induce significant changes in either ABR amplitudes or oscillatory activity, but did increase cortical evoked potentials. Furthermore, in two separate groups of non-implanted animals, we found no evidence that ACEA could reverse behavioural identification of salicylate- or noise-induced tinnitus. Together, these data suggest that while ACEA may be potentially otoprotective, selective CB_1_ agonists are not effective in diminishing the presence of tinnitus or hyperacusis.

## Highlights

• CB_**1**_ agonist (ACEA) effects were assessed in awake guinea pigs following salicylate.
• Salicylate-induced decreases in brainstem response amplitudes were tempered by ACEA.
• Decreases in alpha band oscillations were not evident following salicylate + ACEA.
• ACEA did not eliminate salicylate-induced increases in cortical evoked potentials.
• ACEA failed to prevent or reverse salicylate- or noise-induced tinnitus behaviour.

## 1 Introduction

Cannabis has been used for centuries, both recreationally and for medicinal purposes (Kuddus et al., 2013). In the brain, endogenous cannabinoids are released postsynaptically and act in a retrograde manner on presynaptic cannabinoid receptors, suppressing further release of neurotransmitters (Ohno-Shosaku et al., 2001, Wilson and Nicoll, 2001). There are two types of presynaptically-located cannabinoid receptors, CB_1_ and CB_2_. CB_1_ receptors are predominantly found in the central nervous system, whereas CB_2_ receptors are mainly located on immune cells (see Pertwee, 2006 for a review), although there are also CB_2_ receptors located in the rat cochlear nucleus that could have a functional role (Baek et al., 2008). In the auditory system, CB_1_ receptors are present in the spiral ganglion (Stincic and Hyson, 2011), dorsal and ventral cochlear nucleus (Mailleux and Vanderhaeghen, 1992, Zheng et al., 2007, Zhao et al., 2009), medial nucleus of the trapezoid body (Kushmerick et al., 2004), the inferior colliculus (Moldrich and Wenger, 2000) and the auditory cortex (Eggan and Lewis, 2007). Their presence throughout the auditory system suggests that they play a major role in synaptic regulation (Gerdeman and Lovinger, 2001, Medeiros et al., 2016). However, there are relatively few studies examining how activation of cannabinoid receptors affect the function of the auditory system. We sought to examine whether a highly-selective CB_1_ agonist could be a potential candidate target for attenuating auditory effects caused by sodium salicylate.

Sodium salicylate, the primary metabolite of aspirin in the body (Proost et al., 1983), is commonly used experimentally in animals, as it is known to cause a variety of auditory effects, including tinnitus, hyperacusis and temporary hearing loss (Mongan et al., 1973, Mcfadden et al., 1984, Day et al., 1989, Cazals, 2000, Zhang et al., 2014). Ototoxic effects of salicylate are evident in (1) reduced distortion product otoacoustic emissions (DPOAEs) (Wier et al., 1988, Stolzberg et al., 2011), indicative of reduced outer hair cell activity; 2) a reduction in wave I auditory brainstem responses (ABRs; Berger et al., 2017), indicative of reduced spiral ganglion activity; and 3) reduced amplitude of cochlear compound action potentials (Muller et al., 2003, Stolzberg et al., 2011), indicative of reduced cochlear sensitivity, particularly at high frequencies. Contrastingly, cortical evoked responses are enhanced following salicylate administration (Yang et al., 2007, Sun et al., 2009, Norena et al., 2010, Berger et al., 2017), suggestive of a neural correlate of hyperacusis. Furthermore, reductions in alpha band activity are evident in the auditory cortex of animals following salicylate administration (Stolzberg et al., 2013, Berger et al., 2017), which have been suggested as a correlate of reduced inhibition related to the presence of tinnitus in humans (Weisz et al., 2005, Lorenz et al., 2009).

While there may be differences in the mechanisms underlying the effects of salicylate and noise exposure, which is the primary cause of these conditions in humans, salicylate is particularly useful as it reliably induces tinnitus, hyperacusis and hearing loss in animals when administered at a high dose (e.g. Jastreboff et al., 1988, Lobarinas et al., 2006, Berger et al., 2013, Berger et al., 2017), as opposed to the variability observed following noise exposure. Furthermore, these effects are rapid, evident within 2 h in an animal model (Stolzberg et al., 2011, Berger et al., 2017). It is therefore a useful tool to enable researchers to more easily test whether particular drugs are effective in attenuating neural effects relating to these conditions.

Zheng et al. (2007) demonstrated a significant reduction in the number of ventral cochlear nucleus neurons with CB_1_ receptor expression following salicylate administration in rats. However, Zheng et al. (2010) found that CB_1_ agonists, WIN55,212-2 and CP-55940, were ineffective in eliminating salicylate-induced behavioural deficits, as identified using a conditioned suppression task, and actually induced tinnitus-like behaviour in control rats. In a follow-up study, Zheng et al. (2015) found that a 1:1 ratio of delta-9-tetrahydrocannabinol (THC) and cannabidiol (CBD) administered following noise exposure resulted in a higher number of rats exhibiting tinnitus-like behaviour. However, these cannabinoid agonists are not very selective and may also interact with opioid, vanilloid or muscarinic receptors and this makes the interpretation of their effects more difficult (Pertwee et al., 2010). These are the only two animal studies to date examining the effects of cannabinoid agonists on tinnitus and further work is required to fully understand these effects (Smith and Zheng, 2016), including the use of more selective agonists.

ACEA is a potent, selective full agonist at the CB_1_ receptor and is highly specific, with a 2000-fold higher affinity for the CB_1_ than the CB_2_ receptor. It is a chemically-modified form of the brain-derived cannabinoid anandamide and may be metabolised in a similar way (Hillard et al., 1999). Anandamide is less potent than ACEA and less efficacious (a partial agonist) while another major endocannabinoid in the brain (2-arachidonoyl glycerol) is more efficacious (a full agonist) but less potent. A number of other synthetic cannabinoids were available with high potency and high efficacy, but generally are a lot less selective than ACEA (Pertwee et al., 2010). The physiological effects of CB_1_ agonists have not been studied in the auditory forebrain but a recent study by Toal et al. (2016) demonstrated that CB_1_ receptor knockout mice had deficits in their audiograms above 8 kHz. This suggests a role for CB_1_ receptors in regulating normal hearing, despite their inability to diminish behavioural evidence of tinnitus, and there is a need to further understand the functional significance of the endocannabinoid system in the auditory system (Smith and Zheng, 2016).

We previously demonstrated that salicylate administration significantly reduced wave I ABR amplitudes, increased cortical evoked potentials (EPs) and decreased oscillatory alpha band activity in auditory cortex (Berger et al., 2017). We therefore sought to determine whether these neural changes could be attenuated by ACEA following administration of salicylate in chronically-implanted awake guinea pigs (GPs). We also examined the effects of administering ACEA alone on wave I auditory brainstem amplitudes, cortical evoked potentials and spontaneous oscillatory activity. In a separate group, we assessed whether ACEA could successfully prevent the observation of behavioural evidence of salicylate-induced tinnitus, as assessed using the Preyer reflex gap detection paradigm (Berger et al., 2013). Finally, we examined the effects of ACEA on noise-induced tinnitus-like behaviour in GPs.

## 2 Materials and methods

### 2.1 Animals

All procedures were carried out in accordance with the European Communities Council Directive of 24 November 1986 (86/609/EEC) and the approval of the Animal Welfare and Ethical Review Body at the University of Nottingham, UK. Electrocorticographic (ECoG) recordings were made from a total of 6 male and 3 female tricolour GPs, weighing 500–800 g at the time of implantation (∼2–4 months old). An additional 2 male and 10 female GPs were used for behavioural testing only and were not implanted. Oestrous cycles were not accounted for in female GPs, owing to the fact that, unlike other mammals, they are not easily identified by visual examination in this species when single-sex housed (Stockard and Papanicolaou, 1917). Overall gender and age distributions in the current study were similar to our previous study (Berger et al., 2017).

### 2.2 Electrocorticography (ECoG) array implantation

**Fig. 1:**
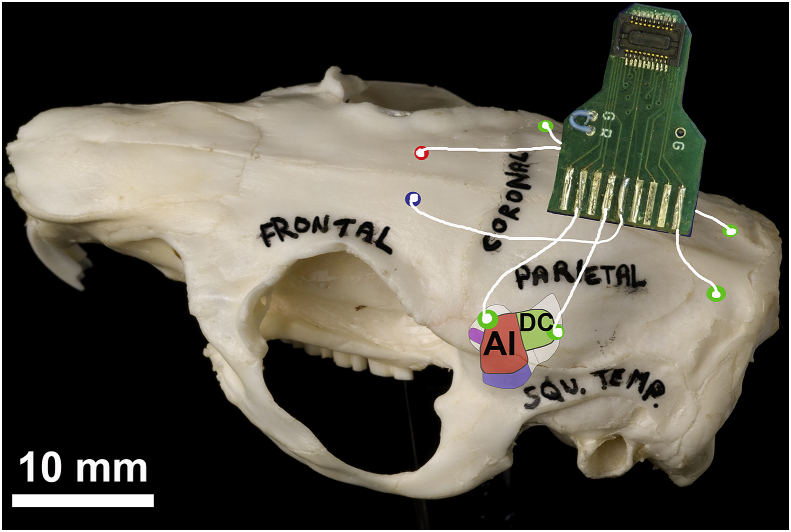
Diagram showing a connector board in relation to a guinea pig skull. The electrode wires and burr holes are shown diagrammatically with the two most rostral holes for the ground and reference (red and blue) and the holes for the two most caudal holes over the cerebellum (green circles at rear). The cortical burr holes (green lateral circles) and are shown in relation to a diagram of the auditory cortical areas which have been superimposed on the side of the skull. The rostral cortical electrode was placed over the dorsorostral belt at the low frequency edge of the primary auditory cortex (AI). The caudal cortical electrode was placed over the caudal belt at the low-frequency edge of the dorsocaudal core area. Electrodes were positioned in relation to local landmarks on the skull, as the lateral suture that forms the border between the parietal and squamous temporal bones runs obliquely across the middle of AI, just before turning medially to become the coronal suture. (For interpretation of the references to colour in this figure legend, the reader is referred to the web version of this article.)

ECoG arrays were prepared and implanted in the same way as described in Berger et al. (2017). Briefly, 8 uninsulated silver wires with silver ball ends were soldered onto a circuit board attached to a Tucker Davis Technologies (TDT: Alachua, FL, USA) zero insertion force (ZIF)-clip connector. Guinea pigs were initially anaesthetised with ketamine (40 mg/kg, i.p.) and xylazine (8 mg/kg, i.p.), and then artificially respired on an isoflurane/O_2_ mixture during the procedure to maintain a constant state of areflexia. Following a midline incision and clearing of muscle and connective tissue, electrodes were placed through burr holes over the rostral and caudal auditory cortex (AC) on each side, with two more positioned over the cerebellum to record ABRs (see Fig. 1). Reference and ground electrodes were linked via a jumper on the electrode board and implanted ∼3 mm rostral to bregma, just off the midline on either side. The rostral and caudal electrodes were putatively placed over the dorsorostral edge of primary AC (abbreviated to rostral) and the dorsocaudal area (abbreviated to caudal), respectively (Grimsley et al., 2012).

After electrode insertion, burr holes and the underside of the electrode board were covered with Kwik-Cast silicone sealant (World Precision Instruments: Hitchin, UK) and dental acrylic. The wound was sutured with Mersilk (Ethicon: Livingston, UK) and covered in antibiotic cream. Cyanoacrylate was applied as an adhesive to the wound around the board. GPs were left for at least 24 h following surgery before baseline ECoG recording.

### 2.3 Recording setup

The ECoG recording setup has been described in detail elsewhere (Berger et al., 2017). Briefly, chronic ABR and ECoG recordings were performed inside a sound-attenuated chamber, using a ZIF-clip digital headstage attached to the GP's implanted electrode. Auditory stimuli were presented free-field via a single ¾-inch tweeter (Tymphany XT19TD00) positioned ∼30 cm above the centre of the cage and were calibrated with two ¼-inch free-field microphones (G.R.A.S. 26AC) placed at either end of the cage prior to each recording session. Recorded ECoG signals were filtered online between 0.5 and 300 Hz for resting state oscillatory activity, 100–5000 Hz for ABRs and 60–300 Hz for EP stimuli. All data were analysed offline with custom-written Matlab (R2014b, MathWorks, MA, USA) scripts. Online data collection was facilitated by either Brainware (software developed by J. Schnupp, University of Oxford, UK) or custom-written Matlab scripts, depending on the types of responses being recorded. Data from electrodes that developed faults were not included in analysis. All experimental conditions (e.g. GP colony, researchers, stimuli, salicylate dose) were the same at those employed in Berger et al. (2017).

### 2.4 ABR stimuli and recording

ABRs were recorded from cerebellar electrodes using custom-written Matlab scripts. Pure tones between 5 and 20 kHz (70 dB SPL, 5 ms duration, 0.1 ms on/off ramp) were presented with 500 repeats in a randomised order, and stimulus polarity was alternated to allow cancellation of stimulus artefact. Offline, recordings with an RMS amplitude >3 absolute deviations above the median were rejected and the remaining data were averaged across repeats. Median absolute deviation was utilised as it has been shown to provide a more robust measure of dispersion than standard deviation when detecting outliers (Leys et al., 2013). Peak-to-trough amplitudes of the largest ABR response (which related to wave I of the subcutaneously-recorded ABR; see Berger et al., 2017) were quantified for each drug treatment and compared to their baseline values. Data from all animals were pooled and treatment effects were assessed statistically with a two-way repeated measures ANOVA and Bonferroni *post-hoc* test. Data were also assessed as changes in ABR amplitude (after treatment values/baseline values) and compared between treatment groups, as well as to the results of Berger et al. (2017). Unpaired *t*-tests were used for determining statistical significance between changes in ABR amplitudes for the different treatment groups (these were assessed as being normally-distributed with the Shapiro-Wilk normality test), and were only applied at the frequency in Berger et al. (2017) where we observed a significant deficit (20 kHz), in order to examine each group independently of one another.

### 2.5 Oscillations and click-evoked responses

Resting state oscillatory activity and click-evoked responses were recorded before and after each drug treatment. Data from all baseline sessions were averaged, consisting of 100 repeats for each session. For resting state activity, each repeat constituted 10 s of recording in silence. Data with an RMS >3 absolute deviations above the median were rejected due to movement artefacts. Power spectral analysis was performed on every 0.5 s sample of this cleaned dataset, resulting in an effective spectral smoothing of 2 Hz, and values were log-transformed in order to express power in decibels (dB) for each frequency. Analysis was completed offline using custom-written Matlab scripts. Statistical analysis comparing baseline resting state activity with that recorded following each drug treatment was performed using a cluster-based permutation test (Maris and Oostenveld, 2007). This involved performing two-tailed, one sample *t*-tests for each frequency. Frequencies with *t*-values greater than a pre-designated threshold (*p* < 0.05) were clustered based on spectral adjacency. All *t*-values within each of these clusters were summed to produce a cluster-level statistic and the maximum summed cluster was used as the test statistic. Data were randomised across conditions for each GP and this method was repeated for every possible permutation of the data, creating a distribution of maximum cluster *t*-values. The actual data were then referenced to this distribution, and clusters which fell outside the 95% confidence interval of the distribution were considered significant.

Neural activity was also recorded in response to short clicks (50 μs), with signals attenuated between 0 and 40 dB SPL of full output (∼100 dB SPL), in 10 dB steps, with an interstimulus interval (ISI) of 1500 ms. Comparisons between baseline and drug treatment data were made using peak-to-trough amplitudes measured within 50 ms following the stimulus.

### 2.6 Drug treatment

Following baseline data collection, we administered intraperitoneal injections of either (1) sodium salicylate (350 mg/kg) 2 h before repeating the above recordings, with ACEA administered 20 min before these recordings (“salicylate + ACEA”, 1 mg/kg, pre-dissolved in anhydrous ethanol at 5 mg/ml; *n* = 5; Tocris, Bristol, UK), or (2) ACEA alone administered 20 min before neural recordings (“ACEA-only”, 1 mg/kg; *n* = 4). ACEA was prepared as a mixture with PEG-35 castor oil (Cremophor EL) and saline solution (0.9% NaCl) at a ratio of 1:1:18 and left to mix for at least 10 min in an ultrasonic bath. Salicylate was injected 2 h before neural recordings, as we have previously demonstrated behavioural effects of salicylate treatment after this time (Berger et al., 2013, Berger et al., 2017). ACEA was administered 20 min before recordings in both groups, to ensure that we captured the peak effect of the ACEA whilst recordings were ongoing, as ACEA has been shown to act quickly following a systemic injection (within 3 min; Hillard et al., 1999) and peak ∼30 min after administration (Hillard et al., 1999, Potenzieri et al., 2008, Reich et al., 2013). Furthermore, at the time we administered ACEA, we would have expected salicylate to already have neural effects (Zhang et al., 2011), so we could examine whether such effects were reversed by ACEA. ACEA was administered at a dose of 1 mg/kg; this dosage has been shown to be effective in treating other conditions, such as motor activity following ischemic stroke (Caltana et al., 2015), while minimising the *in vivo* tetrad of side-effects caused by CB_1_ agonist administration (e.g. Crawley et al., 1993).

### 2.7 Behavioural testing and noise exposure

To determine whether behavioural evidence of tinnitus could successfully be prevented or reversed by administration of ACEA, we performed experiments in two separate groups of non-implanted GPs. These GPs were only used in the behavioural task and no ECoG recordings were performed. The behavioural task employed was the gap detection paradigm originally devised by Turner et al. (2006), adapted for use with the pinna reflex in GPs (Berger et al., 2013), whereby a gap in a continuous background signal preceding a startling stimulus can inhibit the subsequent startle response, resulting in a reduced magnitude of the startle reflex compared with when no gap is present. Following interventions that cause tinnitus, the effect of the gap-induced inhibition is impaired. This is often interpreted as evidence of tinnitus, although it should be noted that others have suggested that the test may instead reflect temporal coding deficits (see Galazyuk and Hebert, 2015 for a review).

In the first group (*n* = 3), we recorded gap detection behavioural responses to broadband noise (BBN) stimuli across six trials per GP, before and 2 h following sodium salicylate administration (350 mg/kg; i.p.). BBN stimuli were selected as we have previously seen reliable gap detection deficits with this background carrier following the same dose of salicylate and at the same time point (Berger et al., 2013, Berger et al., 2017). Furthermore, focussing on one background frequency meant that our data yield and statistical power was much greater than previously, resulting in *n* = 18 gap-induced prepulse inhibition (gap-PPI) trials from 3 GPs at each time point. Sound levels of the background carrier (55, 60, or 70 dB SPL) and startling stimuli (95, 100, or 105 dB SPL) were determined prior to baseline recording with a sound level-dependency test (SLDT; see Berger et al., 2017). Briefly, this involved presenting all different sound level combinations and assessing which combination produced the best level of gap-PPI for each GP, along with a reliable startle response – the optimal levels were then used in baseline and post-treatment trials. Gap duration (50 ms) and number of gap/no gap presentations (10 of each per trial) were identical to regular trials, and each trial lasted ∼2 min. This procedure was performed on a separate day prior to baseline data collection.

ACEA was co-administered 20 min before post-salicylate behavioural testing at the same dose as that used in the ECoG GPs (1 mg/kg; i.p.), with the hypothesis that if ACEA was successful in attenuating neural changes underlying tinnitus, there would be no significant difference between gap/no gap startle ratios before vs after salicylate + ACEA treatment – that is, a gap detection deficit consistent with the presence of tinnitus would not be evident. Behavioural gap detection data across GPs were grouped and statistical significance was assessed using a Wilcoxon matched-pairs signed rank test with an alpha of *p* < 0.05.

In the second group (*n* = 9), we assessed whether ACEA could successfully reverse behavioural evidence of noise-induced tinnitus. Baseline behavioural data were collected for five different background carrier frequencies (BBN, 4–6 kHz, 8–10 kHz, 12–14 kHz, 16–18 kHz), as we have previously found gap detection deficits at a variety of frequencies following the same noise exposure, unlike the uniformity of deficits with salicylate (Coomber et al., 2014). Behavioural sound levels (background: 55, 60 or 70 dB SPL; startling stimulus: 95, 100 or 105 dB SPL) were selected with an SLDT (see above). Each trial consisted of 10 startling stimuli with a gap preceding and 10 startling stimuli with no gap preceding.

Noise exposure was performed in a similar manner as described in Coomber et al. (2014) and Berger et al. (2014). Briefly, GPs were anaesthetised with ketamine (50 mg/kg, i.p.; Fort Dodge Animal Health Ltd, Southampton, UK) and xylazine (10 mg/kg, i.p.; Bayer PLC, Newbury, UK), supplemented with further administrations of a mixture of ketamine and xylazine, in a ratio of 15:2 (i.m.), throughout the procedure. Core body temperature was monitored throughout and maintained at 38 ± 0.5 °C using a homeothermic heating pad (Harvard Apparatus Ltd, Edenbridge, UK) and a rectal probe. Polyethylene tubes (diameter 20 mm) were connected to 25-mm loudspeakers (Peerless DX25). The right one was disconnected and plugged with cotton wool, and placed over the folded right ear, in order to prevent auditory deficits in the right ear. The left speaker was calibrated using a 40BP 0.25-inch pressure condenser microphone, 26AC preamplifier and 12AR power supply (all G.R.A.S, Holte, Denmark) attached to a calibrated 1-mm-diameter probe positioned near the entrance to the ear canal. GPs were placed inside a sound-proof booth and remained there for the duration of the acoustic trauma. The left ear was exposed to narrow band-passed noise bursts (duration of 500 ms and ISI of 200 ms; centre frequency 10 kHz; bandwidth 1 kHz), presented at 116 dB SPL, for 1 h.

Behavioural testing was performed between 7 and 8 weeks following noise exposure in order to determine whether tinnitus was present. Each GP was assessed for tinnitus-like behaviour individually, by comparing sessions recorded after noise exposure with sessions recorded before noise exposure. Tinnitus frequencies were identified using a two-way ANOVA with Bonferroni post-hoc correction (data were normally distributed, as assessed with the Shapiro-Wilk normality test). Average behavioural gap/no gap ratios, at frequencies where a significant deficit was present, were compared to baseline values at the same frequencies, in order to determine the degree of change in behavioural response at the purported tinnitus frequency/frequencies (calculated by dividing the mean of baseline gap/no gap ratios by the mean of post-noise exposure ratios).

GPs were then tested on a further five separate occasions 20 min after administering ACEA (1 mg/kg; i.p). Two GPs with gap detection deficits were reserved for administering ACEA vehicle-only 20 min before behavioural recording. ACEA vehicle was prepared and administered using the same ratio as described earlier for ACEA, but with ethanol substituted for ACEA. The worst session from each time point (baseline, 7–8 weeks and ‘treatment’) was discarded prior to analysis to prevent skewing of the data, for example, on days when GPs were particularly active. Average gap/no gap ratios for the tinnitus frequency/frequencies following treatment were compared to baseline values and Wilcoxon signed rank tests were used to compare changes in gap/no gap ratios across GPs after treatment vs 7–8 weeks, in order to determine whether ACEA or vehicle administration could significantly affect behavioural evidence of noise-induced tinnitus.

## 3 Results

### 3.1 ABR wave I amplitudes

**Fig. 2:**
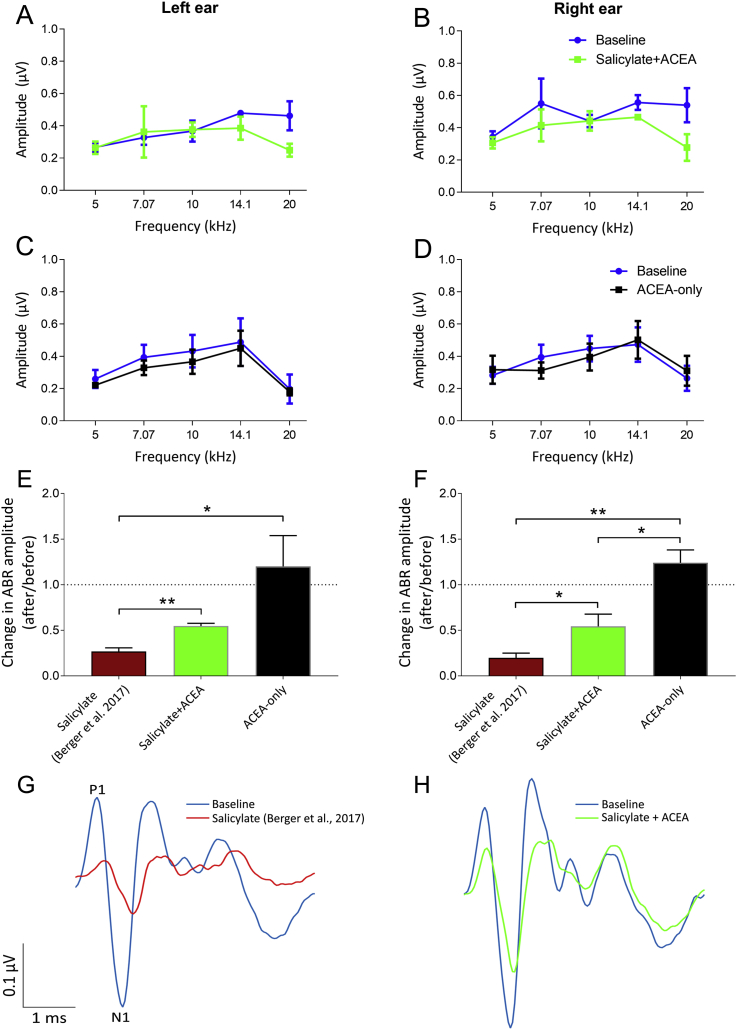
Mean peak-to-trough amplitudes of ABRs, recorded from cerebellar electrodes, for each of the five frequencies recorded. Data are shown for baseline and salicylate + ACEA for left (**A**; *n* = 3) and right (**B**; *n* = 4) ears, as well as baseline and ACEA-only for left (**C**; *n* = 3) and right (**D**; *n* = 4) ears. Change in amplitudes are shown for left (**E**) and right (**F**) ears as after treatment/before treatment ratios, whereby a ratio of 1 indicates no difference in ABR amplitude (dotted line) and a lower ratio highlights a greater reduction in amplitude after treatment. Maroon bars are data from Berger et al. (2017), wherein salicylate was administered without ACEA. Co-administration of ACEA attenuated the salicylate-induced reductions observed previously. Error bars indicate SEM. *p < 0.05; **p < 0.01. **G and H:** Grand averaged ABR waveforms, using data from Berger et al. (2017; **G**) compared to salicylate + ACEA (**H**). These consist of the means across GPs (averaged across repeats) in response to 20 kHz. ACEA attenuated the wave I reductions caused by salicylate, and increases in latencies were not as clear. (For interpretation of the references to colour in this figure legend, the reader is referred to the web version of this article.)

Suprathreshold (70 dB SPL) ABRs were recorded before and 2 h after salicylate administration, as well as 20 min after ACEA administration, for five different frequencies: 5 kHz, 7.07 kHz, 10 kHz, 14.1 kHz and 20 kHz. Previously, we observed a significant decrease in ABR amplitudes following salicylate administration (without co-administration of ACEA; Berger et al., 2017). Average ABR wave I amplitudes for both treatments are shown in Fig. 2. Contrasting with the results of Berger et al. (2017), when ACEA was co-administered with salicylate (salicylate + ACEA group), there was no significant effect of treatment on ABR amplitudes for either the left ear (F_(1,10)_ = 2.83, *p* = 0.12) or the right ear (F_(1,15)_ = 3.88, *p* = 0.07), although a small reduction in wave I amplitudes was evident at 20 kHz for both ears (Fig. 2A and B), albeit not to a statistically significant extent. Furthermore, there was no significant effect of treatment on ABR amplitudes for the left ear (F_(1,10)_ = 3.04, *p* = 0.11) or the right ear (F_(1,15)_ = 0.02, *p* = 0.90) following administration of ACEA by itself (ACEA-only group), thereby suggesting that ACEA did not simply increase ABR wave I amplitudes (Fig. 2C and D).

Changes in ABR amplitudes at 20 kHz were also compared directly with the results of Berger et al. (2017; Fig. 2E and F) and expressed as a ratio calculated as after treatment amplitude/before treatment amplitude, whereby a ratio of 1 indicated no difference in ABR amplitude and lower ratios highlighted a greater reduction in amplitude, as we previously observed a significant deficit at this frequency following salicylate administration alone. For the left ear, in the salicylate + ACEA group, there were some reductions evident in ABR amplitudes, giving a mean ratio (±SEM) of 0.55 (±0.03), but this change was significantly smaller than what we found previously when administering salicylate alone (Berger et al., 2017; maroon bars), *t* (6) = 4.96, *p* = 0.003, wherein the mean ratio (±SEM) was 0.27 (±0.04). For the right ear, this change was again significantly smaller in the salicylate + ACEA group compared to the previous data from Berger et al. (2017), *t* (7) = 2.63, *p* = 0.03, with a mean ratio (±SEM) of 0.54 (±0.13) compared with a mean ratio of 0.20 (±0.05) when salicylate was administered without ACEA. This attenuation of the salicylate-induced reductions of wave I amplitudes by ACEA is further emphasised by Fig. 2G and H, which show left ear grand averages of ABRs in salicylate GPs (from Berger et al., 2017) compared to salicylate + ACEA.

ACEA administered alone resulted in a mean change in ABR ratios at 20 kHz on the left and right of 1.20 (±0.34) and 1.24 (±0.14) respectively. For the left ear, these were significantly higher than the Berger et al. (2017) ratios, *t* (6) = 3.70, *p* = 0.01, but not significantly different than the salicylate + ACEA ratios, *t* (4) = 1.93, *p* = 0.13. For the right ear, these ratios were significantly higher than both the Berger et al. (2017) ratios, *t* (7) = 7.65, *p* = 0.0001, and the salicylate + ACEA ratios, *t* (6) = 3.59, *p* = 0.01. These data further highlight that, although salicylate was still affecting ABR sensitivity, co-administration of ACEA appeared to attenuate the effects of salicylate on ABR amplitudes at the highest frequency we assessed (20 kHz).

### 3.2 ABR wave I latencies

Latencies of the N1 and P1 components of wave I of the ABR were compared for salicylate + ACEA and ACEA alone at all five frequency bands, as we had previously demonstrated a significant increase in P1 and N1 latencies at 20 kHz following salicylate administration (Berger et al., 2017), with increases between 0.12 and 0.16 ms. In the salicylate + ACEA group, there was a general effect of treatment across GPs, with an increase in P1 latencies for the left ear (0.04 ms ± 0.01 SEM; F_(1,10)_ = 4.97, *p* = 0.05) and the right ear (0.06 ms ± 0.01 SEM; F_(1,15)_ = 16.93, *p* = 0.0009), although *post-hoc* testing revealed that there was not one particular frequency with a significant increase in P1 latency. There was also a general increase in N1 latencies across GPs for the left ear (0.04 ms ± 0.01 SEM; F_(1,10)_ = 8.65, *p* = 0.01) and the right ear (0.07 ms ± 0.02 SEM; F_(1,15)_ = 24.66, *p* = 0.0002), although the only particular frequency with a significant increase was 20 kHz for the right ear (t = 3.490, *p* < 0.05), with a mean increase of 0.11 ms (±0.03 ms). In the ACEA-only group, there was no significant effect of treatment on N1 latencies for the left ear (F_(1,10)_ = 2.61, *p* = 0.14) or the right ear (F_(1,15)_ = 2.16, *p* = 0.16), and the same was true for P1 latencies in both the left ear (F_(1,10)_ = 0.02, *p* = 0.89) and the right ear (F_(1,15)_ = 0.01, *p* = 0.92).

### 3.3 Click-evoked cortical EPs

**Fig. 3:**
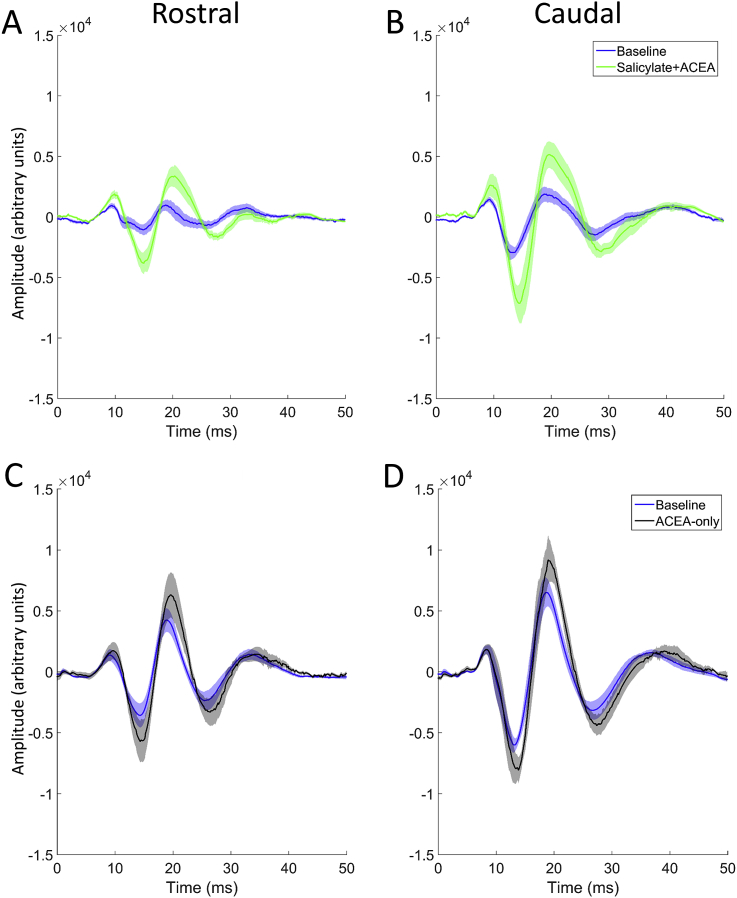
Click-evoked potentials. **A** and **B:** Mean responses across GPs (±SEM; *n* = 5) from rostral AC and caudal AC, recorded during baseline (blue) and after salicylate + ACEA treatment (green). Shading indicates SEM. **C** and **D:** Mean responses across GPs (±SEM; *n* = 4) from rostral AC and caudal AC, recorded during baseline (blue) and after treatment with ACEA alone (black). (For interpretation of the references to colour in this figure legend, the reader is referred to the web version of this article.)

**Fig. 4:**
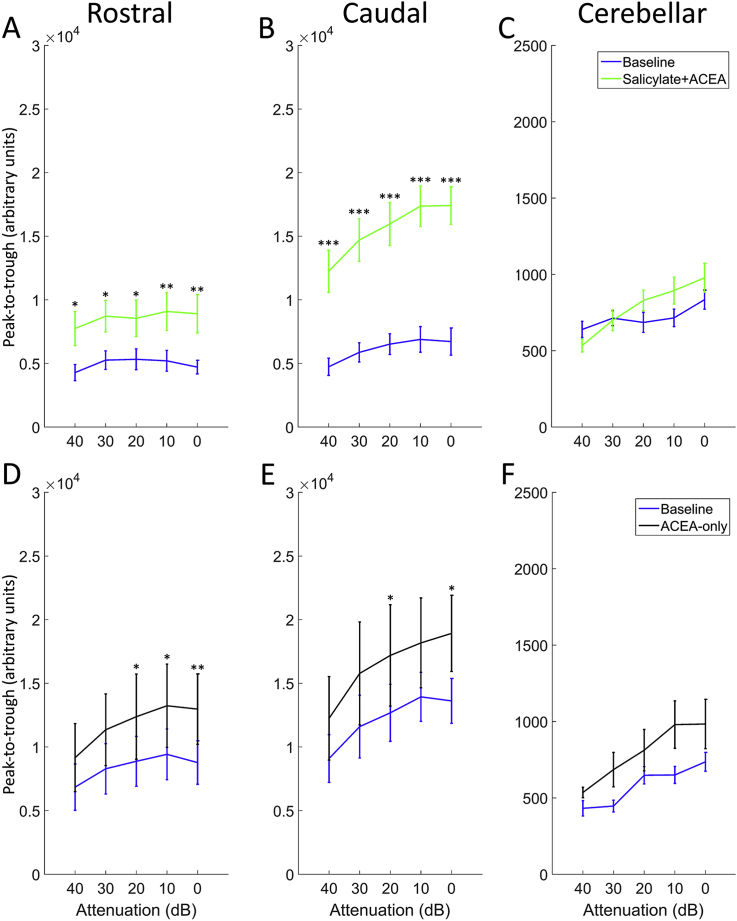
Mean peak-to-trough amplitudes of click-evoked responses across GPs (±SEM) for all attenuations recorded during baseline and after drug administration from rostral AC (**A** and **D**), caudal AC (**B** and **E**) and ABRs (**C** and **F**). *p < 0.05; **p < 0.01; ***p < 0.0001.

Fig. 3 shows EPs in response to 50 μs clicks before vs after the administration of either salicylate + ACEA or ACEA alone, averaged across GPs and recorded with a signal attenuation of 0 dB SPL (approximately 100 dB SPL RMS), while Fig. 4 shows peak-to-trough responses for both groups across all sound levels. Salicylate + ACEA resulted in increases in click-EPs, similar to what we have observed previously with salicylate only (Berger et al., 2017), ranging from 61 to 89% for rostral AC and 145–159% for caudal AC (Fig. 4A and B). All of these increases were significant (*p* < 0.05), with the greatest effects seen at the loudest sound levels. There were no clear or significant changes in cerebellar click-evoked potentials (Fig. 4C). ACEA alone slightly increased EPs for all electrodes (Fig. 4D–F), although there was a considerable degree of variability across GPs and the effect was much lower than that caused by salicylate + ACEA, ranging from 34 to 48% for rostral AC, 30–39% for caudal AC and 24–54% for cerebellar electrodes. Nonetheless, these changes were significant for rostral AC and caudal AC at the loudest sound levels (between 20 and 0 dB attenuation; *p* < 0.05), but were not significant for cerebellar EPs at any sound level. In summary, these data indicate that co-administration of ACEA did not attenuate the increases in click-EPs caused by salicylate, and ACEA alone caused slight increases in EPs.

### 3.4 Cortical oscillatory activity

**Fig. 5:**
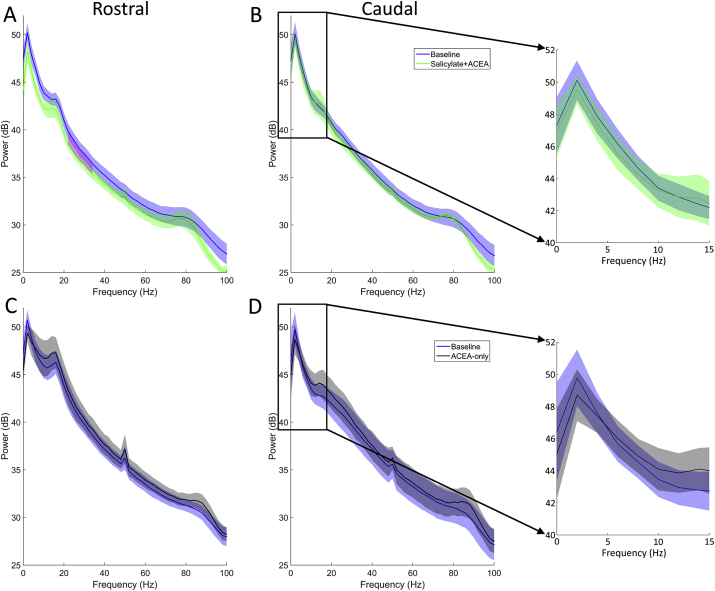
Mean resting-state activity power spectra (±SEM), recorded during baseline and following either salicylate + ACEA (**A**, caudal and **B**, rostral; *n* = 5) or ACEA-only (**C**, caudal and **D**, rostral; *n* = 4). Blue lines indicate baseline activity and blue or black lines indicate activity following drug treatment. Magenta shading between lines in (**A**) highlights significant difference. Inset figures (furthest right) show expanded area where a significant difference was demonstrated previously following salicylate treatment (Berger et al., 2017). (For interpretation of the references to colour in this figure legend, the reader is referred to the web version of this article.)

Data recorded in silence from rostral and caudal electrodes were fast Fourier transformed in order to produce power spectra. Fig. 5 shows mean power spectra averaged across GPs for each group, collected before and after each treatment. Cluster-based permutation analyses were applied to determine statistical significance with an alpha level of *p* < 0.05. In the salicylate + ACEA group, there was a significant decrease in activity between 22 and 34 Hz on the rostral electrodes (Fig. 5A). However, co-administration of ACEA with salicylate did not produce any significant changes in oscillatory activity on the caudal electrodes (Fig. 5B). ACEA administered alone did not cause any significant changes on either the rostral electrodes (Fig. 5C) or the caudal electrodes (Fig. 5D). In summary, the change in oscillatory alpha band activity that we have observed previously following salicylate treatment (Berger et al., 2017) was not present when ACEA was co-administered.

### 3.5 Behavioural responses following salicylate + ACEA administration

**Fig. 6:**
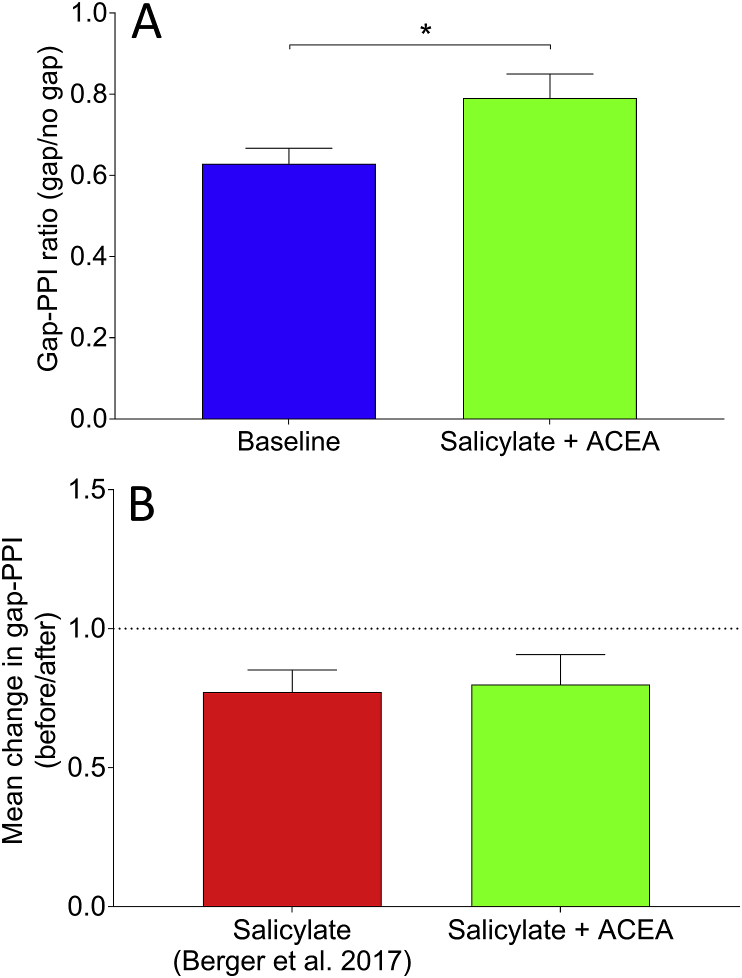
**A**: BBN Behavioural gap detection ratios (gap-PPI) before and after salicylate + ACEA administration, averaged across GPs (*n* = 3 GPs, 18 gap-PPI trials). There was a significant increase in the gap detection ratio of the pinna reflex following co-administration of the two drugs, which highlights that gaps in otherwise continuous background noise were less effective at inhibiting the response following a startling stimulus, consistent with the presence of tinnitus (*p < 0.05). **B:** Mean change from baseline in gap-PPI across GPs (±SEM) following salicylate + ACEA, compared with data from Berger et al. (2017) wherein salicylate was administered without ACEA. A value of 1 would indicate no change in behavioural gap detection ability compared to baseline, whilst higher values would suggest improvement and lower values worsening. There was no significant difference between the two groups.

Fig. 6 shows behavioural responses before and after salicylate + ACEA co-administration, plotted as ratios of gap/no gap, which are indicative of the degree of gap-PPI. Following salicylate + ACEA administration, there was a significant increase in the gap-PPI ratio across GPs (*p* = 0.026), similar to what we have seen previously when administering salicylate alone (Berger et al., 2013, Berger et al., 2017). This indicates that behavioural gap detection ability was impaired following co-administration of both drugs, which is suggestive of the presence of tinnitus and demonstrates that ACEA failed to prevent behavioural identification of salicylate-induced tinnitus. Furthermore, these data were compared directly with data from Berger et al. (2017), wherein salicylate was administered without ACEA. When examining the change in gap-PPI as a function of before salicylate vs after salicylate, calculated for each GP by dividing gap-PPI ratios before drug administration by gap-PPI ratios after, the mean change in gap-PPI ratio (before/after) following salicylate + ACEA was 0.80 (±0.34 SEM), compared to 0.77 (±0.07 SEM) following salicylate without ACEA (data from Berger et al., 2017). This difference was not significant (*U* = 6, *p* = 0.79), further highlighting that ACEA did not attenuate the behavioural effects of salicylate.

### 3.6 Behavioural responses in noise exposed GPs

**Fig. 7:**
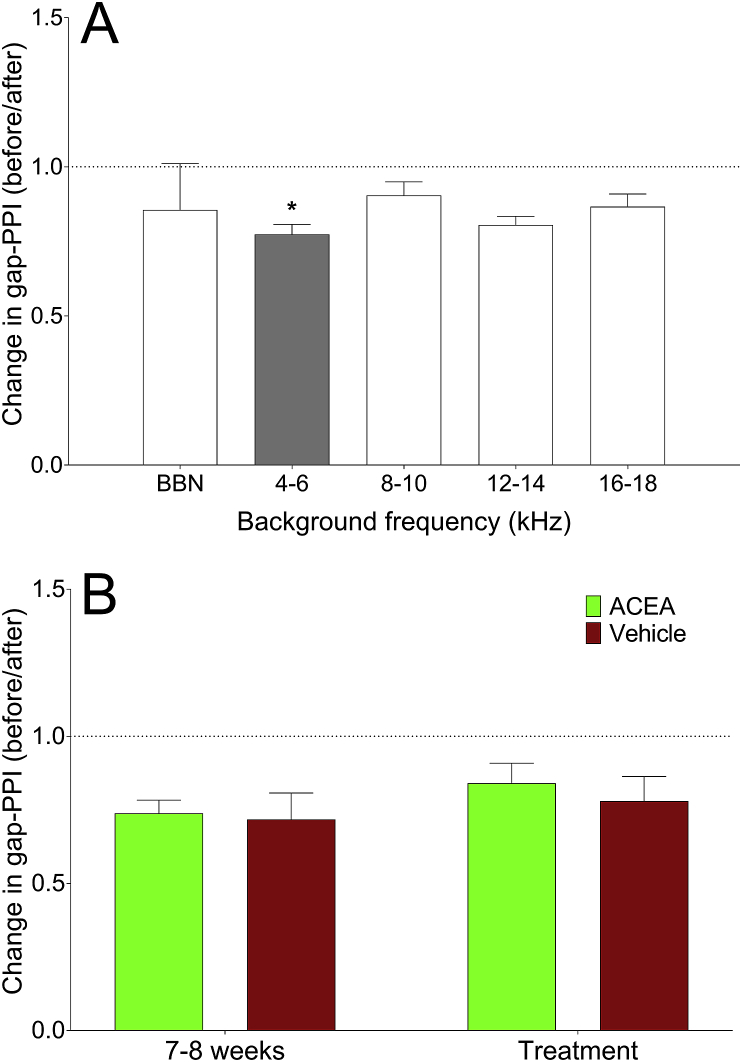
Change in behavioural gap detection ratios (gap-PPI) 7–8 weeks following noise exposure and ACEA administration. **A:** An example of a GP with behavioural evidence of tinnitus. Gap detection ability was significantly worse at the 4–6 kHz background carrier seven-to-eight weeks following noise exposure, compared to baseline (grey bar; *p < 0.05). **B:** Change in gap-PPI at the tinnitus frequency for each time point, compared to baseline (*n* = 6 frequencies for ACEA; *n* = 3 frequencies for vehicle), averaged across GPs. There were no significant changes in behavioural gap detection deficits following either ACEA administration (green bars) or vehicle treatment (maroon bars). (For interpretation of the references to colour in this figure legend, the reader is referred to the web version of this article.)

Behavioural testing for evidence of tinnitus 7–8 weeks following unilateral noise exposure indicated that 7 of the 9 GPs demonstrated significant gap detection deficits at one of more of the frequencies tested, which is proportionally comparable to what we have observed previously with a similar noise exposure (Coomber et al., 2014). In some cases, behavioural gap detection deficits were observed at more than one background frequency. There were significant deficits at 4–6 kHz in four GPs, 12–14 kHz in two GPs, 16–18 kHz in two GPs and BBN in one GP. Fig. 7A shows an example of a GP with a significant deficit at 4–6 kHz, expressed as a change in gap-PPI after noise exposure vs before.

Following behavioural testing for tinnitus, GPs were administered either ACEA or ACEA-vehicle on five separate occasions and their behavioural responses were recorded. Behavioural responses at the suggested tinnitus frequency/frequencies were expressed as an average change in gap-PPI at either the 7–8 week time point or the treatment time point compared to baseline, and data were grouped across GPs. The data for these GPs are shown in Fig. 7B. There was no significant difference in the change in gap-PPI at purported tinnitus frequencies between the 7–8 week time point and the treatment time point, for either ACEA administration (*p* = 0.16; *n* = 6 frequency bands from 5 GPs) or vehicle administration (*p* = 0.25; *n* = 3 frequency bands from 2 GPs), thereby highlighting that ACEA did not significantly affect behavioural evidence of noise-induced tinnitus.

## 4 Discussion

The synthetic cannabinoid agonist, ACEA, attenuated some of the effects that we previously observed with salicylate administration. Namely, it attenuated the reductions in ABR wave I amplitudes and the increases in peak latencies, as well as abolishing decreases in cortical oscillatory activity within the 6–10 Hz band. However, increases in cortical evoked potentials caused by salicylate were not attenuated by co-administration of ACEA. Furthermore, behavioural evidence of both salicylate- and noise-induced tinnitus was still evident following administration of ACEA, suggesting that highly-selective CB_1_ agonists do not prevent the identification of tinnitus or reverse the presence of tinnitus.

### 4.1 Potential otoprotective effects of ACEA

We previously demonstrated that wave I amplitudes were significantly reduced following salicylate administration at 20 kHz (Berger et al., 2017). Following co-administration of ACEA with salicylate, although there were still reductions in wave I amplitudes, these effects were diminished and not significant, suggesting that ACEA had attenuated the ototoxic effects of sodium salicylate. Changes in latencies following salicylate were also attenuated by co-administration of ACEA. Moreover, ACEA administered alone did not cause changes in wave I ABR amplitudes.

Salicylate is known to cause reversible ototoxicity (Stypulkowski, 1990), which may occur through a reduction in outer hair cell electromobility (e.g. Kakehata and Santos-Sacchi, 1996). This would result in a reduction in cochlea sensitivity, which could explain the decreases in wave I ABR amplitudes that we have previously observed. Given that the attenuating effect of ACEA that we have observed here is purely phenomenological, we can only speculate as to the underlying mechanisms. There is recent evidence that CB_1_ receptors are present in the cochlea, although the CB_2_ receptors appeared more prevalent (Martin-Saldana et al., 2016). Furthermore in the same study, following cisplatin treatment, which produces ototoxicity, CB_1_ receptor expression was downregulated in spiral ganglion neurons. Jeong et al. (2007) also demonstrated that a CB_1_ agonist, HU-210, could prevent apoptosis of hair cells following cisplatin treatment. Recent data suggest that salicylate may kill spiral ganglion neurons over the course of several days as a result of enhancing activation of NMDA receptors (Deng et al., 2013).

Cannabinoids have been shown to prevent neurotoxicity caused by over-activation of NMDARs in other conditions (e.g. El-Remessy et al., 2003), as they can act in a similar manner to non-competitive NMDAR antagonists (Feigenbaum et al., 1989). Therefore, by activating CB_1_ receptors it is possible that we attenuated processes that would lead to the apoptotic effects of salicylate that have previously been demonstrated (Wei et al., 2010, Feng et al., 2011). All of the above, combined with the fact that mice missing CB_1_ receptors possess poorer hearing thresholds than wild-type mice (Toal et al., 2016), suggests an important role for CB_1_ receptors in protecting and maintaining normal hearing function. Clearly further work is required in order to determine the specific underlying mechanisms by which activation of CB_1_ receptors may attenuate damage caused by salicylate.

Furthermore, it would be of interest to determine whether ACEA is successful at attenuating damage to the periphery caused by noise exposure. Given that we did not examine ABRs in our noise exposed GPs following ACEA administration, we are unable to determine whether this was the case in our GPs. Recent work by Zheng et al. (2015) did not find a difference in ABR thresholds between noise-exposed rats administered delta-9-THC and CBD, so the effects seen here may only be apparent in salicylate-induced hearing loss. However, absolute ABR thresholds do not appear to be as sensitive as ABR amplitudes at suprathreshold sound levels in measuring the extent of damage at the periphery (Kujawa and Liberman, 2009). It is also possible that administering a CB_1_ agonist prior to an acoustic insult (as opposed to after) may prevent subsequent damage, rather than reversing already-induced damage. If this was the case, however, then the impact of such a treatment would be limited in humans, as it would not reverse pre-existing damage.

### 4.2 No effect on cortical evoked potentials

Despite the attenuation of effects observed at the periphery, co-administration of ACEA failed to attenuate the effects of salicylate on cortical evoked potentials, and in fact significantly increased click-evoked potentials at the highest sound levels when administered alone. To our knowledge, this is the first demonstration of effects of a CB_1_ agonist on auditory cortex EP amplitudes, although enhancements in EPs have previously been demonstrated in the hippocampus with the CB_1_ agonist WIN55,212-2 (Dissanayake et al., 2008). This also suggests a central mechanism for salicylate-induced hyperacusis, independent of peripheral activity, an idea supported by others (Stolzberg et al., 2012b, Chen et al., 2013, Radziwon et al., 2017). There is clear evidence that salicylate administered systemically enters both the cochlear perilymph and cerebrospinal fluid (Jastreboff et al., 1986), thereby directly affecting auditory areas of the central nervous system (see Stolzberg et al., 2012a for a review). Therefore, it is not inconsistent that we observed different effects on wave I ABR activity to cortical evoked activity, as both drugs would likely act peripherally and centrally.

CB_1_ receptors are present in cholecystokinin-expressing large basket cells in the neocortex (e.g. Bodor et al., 2005), which are inhibitory interneurons. In these neurons, CB_1_ receptors are located presynaptically, which has a net effect of depressing inhibition (see Allene et al., 2015 for a review). This is evidenced by the fact that administration of a CB_1_ agonist suppresses inhibition in layers 2/3 of the auditory cortex (Trettel and Levine, 2002). Therefore, it is perhaps not surprising that EP amplitudes were slightly enhanced by cannabinoids. Consistent with this finding, Guy and Flint (2004) reported hyperacusis in humans as an adverse side effect of oral administration of CBD:THC prepared as a 1:1 ratio.

### 4.3 Decreases in alpha band oscillations absent following co-administration of salicylate with ACEA

We previously demonstrated a significant reduction in alpha band activity in caudal AC following salicylate administration (Berger et al., 2017). Following co-administration of ACEA, significant changes in alpha band activity were not present in the current study. If a reduction in alpha band activity is a neural correlate of tinnitus, then this would suggest that ACEA was successfully eliminating the neural underpinnings of tinnitus. However, Zheng et al., 2010, Zheng et al., 2015 found that CB_1_ agonists were ineffective at reducing behavioural evidence of tinnitus following either salicylate or noise exposure. Furthermore, we found that ACEA was unsuccessful in preventing behavioural evidence of salicylate-induced tinnitus or reversing behavioural evidence of noise-induced tinnitus, which is consistent with the results of Zheng et al., 2010, Zheng et al., 2015. Therefore, even if a reduction in alpha band activity is an underlying contributor to the tinnitus percept, given that a cannabinoid agonist did not abolish behavioural evidence of tinnitus, but did prevent changes in alpha band activity following salicylate administration, it appears unlikely that it is necessary for generating the phantom percept.

One limitation of the current study is that the effect of vehicle administration on behavioural deficits following noise exposure was only examined in two guinea pigs (one of which had putative tinnitus at two background frequencies, resulting in *n* = 3 tinnitus frequencies before vs after vehicle). Therefore, although we did not observe any effect, the statistical test in this case would have likely been underpowered. However, behavioural deficits following noise exposure were also unchanged following ACEA administration (with *n* = 6 putative tinnitus frequencies), which made the comparison with vehicle GPs less crucial. As a result, we do not feel that the low number of vehicle controls significantly affects the conclusions we can draw from the data.

Significant decreases in low gamma activity were evident in rostral AC following co-administration of salicylate and ACEA. We demonstrated previously that both a vehicle and a salicylate injection were capable of producing increases in gamma activity in this same region (Berger et al., 2017), which we suggested may be a result of stress associated with the injection. The decreases in gamma we have observed here therefore suggest that the ACEA injection may actually reduce stress in these GPs. This idea, however, is not supported by the fact that we did not see any significant changes following an injection of ACEA alone. Therefore, there could be an interaction effect between salicylate and ACEA that results in this change in low gamma activity.

Whilst we did not observe such a reduction in gamma band activity with administration of ACEA only, previous studies have demonstrated that another CB_1_ agonist, CP-55940, reduced gamma oscillations in other areas, including in the hippocampus and entorhinal cortex (Robbe et al., 2006, Hajos et al., 2008) and prefrontal cortex (Kucewicz et al., 2011). Conversely, Morgan et al. (2008) failed to find a difference in neural oscillations in the medial entorhinal cortex following application of the CB_1_ agonist arachidonylcyclopropylamide (ACPA) *in vitro*. Nonetheless, clearly further research is required to properly understand the mechanisms underlying changes in oscillatory activity observed here.

In summary, ACEA appeared to attenuate salicylate-induced reductions in wave I of the ABR and alpha-band neural oscillations, but did not attenuate salicylate-induced increases in cortical evoked potentials or affect tinnitus-like behaviour. This suggests that ACEA may be potentially otoprotective, and that hyperacusis-like activity may occur independent of peripheral insult, but CB_1_ agonists are not suitable as a potential treatment for tinnitus. Further research is necessary to determine whether ACEA can effectively attenuate peripheral damage caused by excessive noise exposure.

## References

[bib1] Allene C., Lourenco J., Bacci A. (2015). The neuronal identity bias behind neocortical GABAergic plasticity. Trends Neurosci..

[bib2] Baek J.H., Zheng Y., Darlington C.L., Smith P.F. (2008). Cannabinoid CB2 receptor expression in the rat brainstem cochlear and vestibular nuclei. Acta Otolaryngol..

[bib3] Berger J.I., Coomber B., Wallace M.N., Palmer A.R. (2017). Reductions in cortical alpha activity, enhancements in neural responses and impaired gap detection caused by sodium salicylate in awake Guinea pigs. Eur. J. Neurosci..

[bib4] Berger J.I., Coomber B., Shackleton T.M., Palmer A.R., Wallace M.N. (2013). A novel behavioural approach to detecting tinnitus in the Guinea pig. J. Neurosci. Methods.

[bib5] Berger J.I., Coomber B., Wells T.T., Wallace M.N., Palmer A.R. (2014). Changes in the response properties of inferior colliculus neurons relating to tinnitus. Front. Neurol..

[bib6] Bodor A.L., Katona I., Nyiri G., Mackie K., Ledent C., Hajos N., Freund T.F. (2005). Endocannabinoid signaling in rat somatosensory cortex: laminar differences and involvement of specific interneuron types. J. Neurosci..

[bib7] Caltana L., Saez T.M., Aronne M.P., Brusco A. (2015). Cannabinoid receptor type 1 agonist ACEA improves motor recovery and protects neurons in ischemic stroke in mice. J. Neurochem..

[bib8] Cazals Y. (2000). Auditory sensori-neural alterations induced by salicylate. Prog. Neurobiol..

[bib9] Chen G.D., Stolzberg D., Lobarinas E., Sun W., Ding D., Salvi R. (2013). Salicylate-induced cochlear impairments, cortical hyperactivity and re-tuning, and tinnitus. Hear Res..

[bib10] Coomber B., Berger J.I., Kowalkowski V.L., Shackleton T.M., Palmer A.R., Wallace M.N. (2014). Neural changes accompanying tinnitus following unilateral acoustic trauma in the Guinea pig. Eur. J. Neurosci..

[bib11] Crawley J.N., Corwin R.L., Robinson J.K., Felder C.C., Devane W.A., Axelrod J. (1993). Anandamide, an endogenous ligand of the cannabinoid receptor, induces hypomotility and hypothermia in vivo in rodents. Pharmacol. Biochem. Behav..

[bib12] Day R.O., Graham G.G., Bieri D., Brown M., Cairns D., Harris G., Hounsell J., Platthepworth S., Reeve R., Sambrook P.N., Smith J. (1989). Concentration-response relationships for salicylate-induced ototoxicity in normal volunteers. Brit. J. Clin. Pharm..

[bib13] Deng L., Ding D., Su J., Manohar S., Salvi R. (2013). Salicylate selectively kills cochlear spiral ganglion neurons by paradoxically up-regulating superoxide. Neurotox. Res..

[bib14] Dissanayake D.W., Zachariou M., Marsden C.A., Mason R. (2008). Auditory gating in rat hippocampus and medial prefrontal cortex: effect of the cannabinoid agonist WIN55,212-2. Neuropharmacology.

[bib15] Eggan S.M., Lewis D.A. (2007). Immunocytochemical distribution of the cannabinoid CB1 receptor in the primate neocortex: a regional and laminar analysis. Cereb. Cortex.

[bib16] El-Remessy A.B., Khalil I.E., Matragoon S., Abou-Mohamed G., Tsai N.J., Roon P., Caldwell R.B., Caldwell R.W., Green K., Liou G.I. (2003). Neuroprotective effect of(-)Delta(9)-tetrahydrocannabinol and cannabidiol in N-methyl-D-aspartate-induced retinal neurotoxicity - involvement of peroxynitrite. Am. J. Pathol..

[bib17] Feigenbaum J.J., Bergmann F., Richmond S.A., Mechoulam R., Nadler V., Kloog Y., Sokolovsky M. (1989). Nonpsychotropic cannabinoid acts as a functional N-Methyl-D-Aspartate receptor blocker. P Natl. Acad. Sci. U. S. A..

[bib18] Feng H., Yin S.H., Tang A.Z., Tan S.H. (2011). Salicylate initiates apoptosis in the spiral ganglion neuron of Guinea pig cochlea by activating caspase-3. Neurochem. Res..

[bib19] Galazyuk A., Hebert S. (2015). Gap-prepulse inhibition of the acoustic startle reflex (GPIAS) for tinnitus assessment: current status and future directions. Front. Neurol..

[bib20] Gerdeman G., Lovinger D.M. (2001). CB1 cannabinoid receptor inhibits synaptic release of glutamate in rat dorsolateral striatum. J. Neurophysiol..

[bib21] Grimsley J.M., Shanbhag S.J., Palmer A.R., Wallace M.N. (2012). Processing of communication calls in Guinea pig auditory cortex. PLoS One.

[bib22] Guy G.W., Flint M.E. (2004). A single centre, placebo-controlled, four period, crossover, tolerability study assessing, pharmacodynamic effects, pharmacokinetic characteristics and cognitive profiles of a single Dose of three formulations of cannabis based medicine extracts (CBMEs) (GWPD9901), plus a two period tolerability study comparing pharmacodynamic effects and pharmacokinetic characteristics of a single Dose of a cannabis based medicine extract given via two administration routes (GWPD9901 EXT). J. Cannabis Ther..

[bib23] Hajos M., Hoffmann W.E., Kocsis B. (2008). Activation of cannabinoid-1 receptors disrupts sensory gating and neuronal oscillation: relevance to schizophrenia. Biol. Psychiatry.

[bib24] Hillard C.J., Manna S., Greenberg M.J., Dicamelli R., Ross R.A., Stevenson L.A., Murphy V., Pertwee R.G., Campbell W.B. (1999). Synthesis and characterization of potent and selective agonists of the neuronal cannabinoid receptor (CB1). J. Pharmacol. Exp. Ther..

[bib25] Jastreboff P.J., Hansen R., Sasaki P.G., Sasaki C.T. (1986). Differential uptake of salicylate in serum, cerebrospinal fluid, and perilymph. Arch. Otolaryngol. Head Neck Surg..

[bib26] Jastreboff P.J., Brennan J.F., Coleman J.K., Sasaki C.T. (1988). Phantom auditory sensation in rats - an animal-model for tinnitus. Behav. Neurosci..

[bib27] Jeong H.J., Kim S.J., Moon P.D., Kim N.H., Kim J.S., Park R.K., Kim M.S., Park B.R., Jeong S., Um J.Y., Kim H.M., Hong S.H. (2007). Antiapoptotic mechanism of cannabinoid receptor 2 agonist on cisplatin-induced apoptosis in the HEI-OC1 auditory cell line. J. Neurosci. Res..

[bib28] Kakehata S., Santos-Sacchi J. (1996). Effects of salicylate and lanthanides on outer hair cell motility and associated gating charge. J. Neurosci..

[bib29] Kucewicz M.T., Tricklebank M.D., Bogacz R., Jones M.W. (2011). Dysfunctional prefrontal cortical network activity and interactions following cannabinoid receptor activation. J. Neurosci..

[bib30] Kuddus M., Ginawi I.A.M., Al-Hazimi A. (2013). Cannabis sativa: an ancient wild edible plant of India. Emir J. Food Agric..

[bib31] Kujawa S.G., Liberman M.C. (2009). Adding insult to injury: cochlear nerve degeneration after "temporary" noise-induced hearing loss. J. Neurosci..

[bib32] Kushmerick C., Price G.D., Taschenberger H., Puente N., Renden R., Wadiche J.I., Duvoisin R.M., Grandes P., von Gersdorff H. (2004). Retroinhibition of presynaptic Ca2+ currents by endocannabinoids released via postsynaptic mGluR activation at a calyx synapse. J. Neurosci..

[bib33] Leys C., Ley C., Klein O., Bernard P., Licata L. (2013). Detecting outliers: Do not use standard deviation around the mean, use absolute deviation around the median. J. Exp. Soc. Psychol..

[bib34] Lobarinas E., Yang G., Sun W., Ding D., Mirza N., Dalby-Brown W., Hilczmayer E., Fitzgerald S., Zhang L.Y., Salvi R. (2006). Salicylate- and quinine-induced tinnitus and effects of memantine. Acta Oto-Laryngol..

[bib35] Lorenz I., Muller N., Schlee W., Hartmann T., Weisz N. (2009). Loss of alpha power is related to increased gamma synchronization-A marker of reduced inhibition in tinnitus?. Neurosci. Lett..

[bib36] Mailleux P., Vanderhaeghen J.J. (1992). Distribution of neuronal cannabinoid receptor in the adult rat brain: a comparative receptor binding radioautography and in situ hybridization histochemistry. Neuroscience.

[bib37] Maris E., Oostenveld R. (2007). Nonparametric statistical testing of EEG- and MEG-data. J. Neurosci. Methods.

[bib38] Martin-Saldana S., Trinidad A., Ramil E., Sanchez-Lopez A.J., Coronado M.J., Martinez-Martinez E., Garcia J.M., Garcia-Berrocal J.R., Ramirez-Camacho R. (2016). Spontaneous cannabinoid receptor 2 (CB2) expression in the cochlea of adult albino rat and its up-regulation after cisplatin treatment. PLoS One.

[bib39] Mcfadden D., Plattsmier H.S., Pasanen E.G. (1984). Aspirin-induced hearing-loss as a model of sensorineural hearing-loss. Hear. Res..

[bib40] Medeiros P., de Freitas R.L., Silva M.O., Coimbra N.C., Melo-Thomas L. (2016). CB1 cannabinoid receptor-mediated anandamide signaling mechanisms of the inferior colliculus modulate the haloperidol-induced catalepsy. Neuroscience.

[bib41] Moldrich G., Wenger T. (2000). Localization of the CB1 cannabinoid receptor in the rat brain. An immunohistochemical study. Peptides.

[bib42] Mongan E., Kelly P., Nies K., Porter W.W., Paulus H.E. (1973). Tinnitus as an indication of therapeutic serum salicylate levels. JAMA.

[bib43] Morgan N.H., Stanford I.M., Woodhall G.L. (2008). Modulation of network oscillatory activity and GABAergic synaptic transmission by CB1 cannabinoid receptors in the rat medial entorhinal cortex. Neural Plast..

[bib44] Muller M., Klinke R., Arnold W., Oestreicher E. (2003). Auditory nerve fibre responses to salicylate revisited. Hear. Res..

[bib45] Norena A.J., Moffat G., Blanc J.L., Pezard L., Cazals Y. (2010). Neural changes in the auditory cortex of awake Guinea pigs after two tinnitus inducers: salicylate and acoustic trauma. Neuroscience.

[bib46] Ohno-Shosaku T., Maejima T., Kano M. (2001). Endogenous cannabinoids mediate retrograde signals from depolarized postsynaptic neurons to presynaptic terminals. Neuron.

[bib47] Pertwee R.G. (2006). The pharmacology of cannabinoid receptors and their ligands: an overview. Int. J. Obes..

[bib48] Pertwee R.G., Howlett A.C., Abood M.E., Alexander S.P.H., Di Marzo V., Elphick M.R., Greasley P.J., Hansen H.S., Kunos G., Mackie K., Mechoulam R., Ross R.A. (2010). International union of basic and clinical pharmacology. LXXIX. Cannabinoid receptors and their ligands: beyond CB1 and CB2. Pharmacol. Rev..

[bib49] Potenzieri C., Brink T.S., Pacharinsak C., Simone D.A. (2008). Cannabinoid modulation of cutaneous Adelta nociceptors during inflammation. J. Neurophysiol..

[bib50] Proost J.H., Van Imhoff G.W., Wesseling H. (1983). Plasma levels of acetylsalicylic acid and salicylic acid after oral ingestion of plain and buffered acetylsalicylic acid in relation to bleeding time and thrombocyte function. Pharm. Weekbl. Sci..

[bib51] Radziwon K., Holfoth D., Lindner J., Kaier-Green Z., Bowler R., Urban M., Salvi R. (2017). Salicylate-induced hyperacusis in rats: Dose- and frequency-dependent effects. Hear Res..

[bib52] Reich C.G., Iskander A.N., Weiss M.S. (2013). Cannabinoid modulation of chronic mild stress-induced selective enhancement of trace fear conditioning in adolescent rats. J. Psychopharmacol..

[bib53] Robbe D., Montgomery S.M., Thome A., Rueda-Orozco P.E., McNaughton B.L., Buzsaki G. (2006). Cannabinoids reveal importance of spike timing coordination in hippocampal function. Nat. Neurosci..

[bib54] Smith P.F., Zheng Y. (2016). Cannabinoids, cannabinoid receptors and tinnitus. Hear Res..

[bib55] Stincic T.L., Hyson R.L. (2011). The localization and physiological effects of cannabinoid receptor 1 in the brain stem auditory system of the chick. Neuroscience.

[bib56] Stockard C.R., Papanicolaou G.N. (1917). The existence of a typical oestrous cycle in the Guinea-pig—with a study of its histological and physiological changes. Am. J. Anat..

[bib57] Stolzberg D., Chen G.D., Allman B.L., Salvi R.J. (2011). Salicylate-induced peripheral auditory changes and T'onotopic reorganization of auditory cortex. Neuroscience.

[bib58] Stolzberg D., Salvi R.J., Allman B.L. (2012). Salicylate toxicity model of tinnitus. Front. Syst. Neurosci..

[bib59] Stolzberg D., Chrostowski M., Salvi R.J., Allman B.L. (2012). Intracortical circuits amplify sound-evoked activity in primary auditory cortex following systemic injection of salicylate in the rat. J. Neurophysiol..

[bib60] Stolzberg D., Hayes S.H., Kashanian N., Radziwon K., Salvi R.J., Allman B.L. (2013). A novel behavioral assay for the assessment of acute tinnitus in rats optimized for simultaneous recording of oscillatory neural activity. J. Neurosci. Methods.

[bib61] Stypulkowski P.H. (1990). Mechanisms of salicylate ototoxicity. Hear Res..

[bib62] Sun W., Lu J., Stolzberg D., Gray L., Deng A., Lobarinas E., Salvi R.J. (2009). Salicylate increases the gain of the central auditory system. Neuroscience.

[bib63] Toal K.L., Radziwon K.E., Holfoth D.P., Xu-Friedman M.A., Dent M.L. (2016). Audiograms, gap detection thresholds, and frequency difference limens in cannabinoid receptor 1 knockout mice. Hear. Res..

[bib64] Trettel J., Levine E.S. (2002). Cannabinoids depress inhibitory synaptic inputs received by layer 2/3 pyramidal neurons of the neocortex. J. Neurophysiol..

[bib65] Turner J.G., Brozoski T.J., Bauer C.A., Parrish J.L., Myers K., Hughes L.F., Caspary D.M. (2006). Gap detection deficits in rats with tinnitus: a potential novel screening tool. Behav. Neurosci..

[bib66] Wei L., Ding D., Salvi R. (2010). Salicylate-induced degeneration of cochlea spiral ganglion neurons-apoptosis signaling. Neuroscience.

[bib67] Weisz N., Moratti S., Meinzer M., Dohrmann K., Elbert T. (2005). Tinnitus perception and distress is related to abnormal spontaneous brain activity as measured by magnetoencephalography. Plos Med..

[bib68] Wier C.C., Pasanen E.G., Mcfadden D. (1988). Partial dissociation of spontaneous otoacoustic emissions and Distortion products during aspirin use in humans. J. Acoust. Soc. Am..

[bib69] Wilson R.I., Nicoll R.A. (2001). Endogenous cannabinoids mediate retrograde signalling at hippocampal synapses. Nature.

[bib70] Yang G., Lobarinas E., Zhang L.Y., Turner J., Stolzberg D., Salvi R., Sun W. (2007). Salicylate induced tinnitus: behavioral measures and neural activity in auditory cortex of awake rats. Hear. Res..

[bib71] Zhang C., Flowers E., Li J.X., Wang Q.J., Sun W. (2014). Loudness perception affected by high doses of salicylate-A behavioral model of hyperacusis. Behav. Brain Res..

[bib72] Zhang X., Yang P., Cao Y., Qin L., Sato Y. (2011). Salicylate induced neural changes in the primary auditory cortex of awake cats. Neuroscience.

[bib73] Zhao Y., Rubio M.E., Tzounopoulos T. (2009). Distinct functional and anatomical architecture of the endocannabinoid system in the auditory brainstem. J. Neurophysiol..

[bib74] Zheng Y., Reid P., Smith P.F. (2015). Cannabinoid CB1 receptor agonists Do not Decrease, but may increase acoustic trauma-induced tinnitus in rats. Front. Neurol..

[bib75] Zheng Y., Baek J.H., Smith P.F., Darlington C.L. (2007). Cannabinoid receptor down-regulation in the ventral cochlear nucleus in a salicylate model of tinnitus. Hear Res..

[bib76] Zheng Y., Stiles L., Hamilton E., Smith P.F., Darlington C.L. (2010). The effects of the synthetic cannabinoid receptor agonists, WIN55,212-2 and CP55,940, on salicylate-induced tinnitus in rats. Hear Res..

